# Structural Characterization of the Rhenium(V) Oxo Complex of Mercaptoacetyltriglyclne in its Dianionic Form

**DOI:** 10.1155/MBD.1995.105

**Published:** 1995

**Authors:** Lory Hansen, Andrew Taylor, Luigi G. Marzilli

**Affiliations:** 1 Emory University Department of Radiology Atlanta Georgia 30322 USA; 2 Emory University Department of Chemistry Atlanta Georgia 30322 USA

## Abstract

Dianionic [MO(MAG_3_)]^2−^(MAG_3_ = penta-anionic form of mercaptoacetyltriglycine, M = ^186^Re, ^99m^Tc) complexes have important applications in nuclear medicine. In vivo the complexes have a deprotonated carboxyl group that is important to their biodistribution. The solid-state structures of ^99^Tc and Re complexes with mercaptoacetyltriglycine reported previously are monoanions with protonated carboxyl groups. In the present work, we report the preparation and X-ray crystal structure of Na_2_[ReO(MAG_3_)]·5H_2_O (1), which contains the physiologically relevant dianion. The dianion is a distorted square pyramid with the nitrogen and sulphur donor atoms forming the base and the oxo ligand at the apex. The terminal carboxyl group is deprotonated, uncoordinated and has a *syn* orientation with respect to the oxo ligand. The *syn* conformation of the dianion in 1 differs in conformation from the *anti*-monoanion in [Bu_4_N][ReO(MAG_3_H)] but is similar to the *syn*-monoanion in [Ph_4_P][^99^TcO(MAG_3_ H)].


### STRUCTURAL CHARACTERIZATION OF THE RHENIUM(V) OXO COMPLEX OF MERCAPTOACETYLTRIGLYCINE IN ITS DIANIONIC FORM

Lory Hansen,[1*] Andrew Taylor, Jr.[1] and Luigi G. Marzilli[2]

Emory University,
[1] Department of Radiology and [2] Department of Chemistry,
Atlanta, Georgia 30322, USA

### ABSTRACT

Dianionic $[MO(MAG_3)]^{2-}$ ($MAG_3$ = penta-anionic form of mercaptoacetyltriglycine, M = $^{186}Re$, $^{99m}Tc$) complexes have important applications in nuclear medicine. In vivo the complexes have a deprotonated carboxyl group that is important to their biodistribution. The solid-state structures of $^{99}Tc$ and Re complexes with mercaptoacetyltriglycine reported previously are monoanions with protonated carboxyl groups. In the present work, we report the preparation and X-ray crystal structure of $Na_2[ReO(MAG_3)]\cdot 5H_2O$ (**1**), which contains the physiologically relevant dianion. The dianion is a distorted square pyramid with the nitrogen and sulphur donor atoms forming the base and the oxo ligand at the apex. The terminal carboxyl group is deprotonated, uncoordinated and has a *syn* orientation with respect to the oxo ligand. The *syn* conformation of the dianion in **1** differs in conformation from the *anti*-monoanion in $[Bu_4N][ReO(MAG_3H)]$ but is similar to the *syn* -monoanion in $[Ph_4P][^{99}TcO(MAG_3H)]$.

### INTRODUCTION

Complexes of $^{99m}Tc$ and $^{186}Re$ radionuclides and mercaptoacetyltriglycine have important applications in nuclear medicine.[1-3] The $^{99m}Tc$ derivative accounts for 40% of the estimated 420,000 renal scans performed annually in the United States.[4] However, the relationship between the physical properties of these small coordination complexes and their biodistribution is not completely understood. This is due in part to an inadequate knowledge of their in vivo chemical structure. The crystal structures of $[Ph_4As][^{99}TcO(MAG_3H)]$[5,6] ($^{99}Tc$, $t_{1/2}$ = 2.12 x $10^5$ years) and $[Bu_4N][ReO(MAG_3H)]$[7] ($MAG_3H$ = tetra-anionic form of mercaptoacetyltriglycyine) have been determined. Unfortunately, fully refined structural details have not been presented. Furthermore, in both these mono-anionic solid-state structures the carboxyl groups are protonated, whereas the species that is present in solution at physiological pH is dianionic with a deprotonated carboxyl group. In addition, the orientation of the carboxyl group with respect to the oxo ligand is different in $[Ph_4As][^{99}TcO(MAG_3H)]$[5,6] and $[Bu_4N][ReO(MAG_3H)]$.[7] The orientation is *syn* in $[Ph_4As][^{99}TcO(MAG_3H)]$ and *anti* in $[Bu_4N][ReO(MAG_3H)]$. Each of these factors limits the usefulness of the structural results in establishing structure-(bio)distribution relationships.

One of our goals has been to crystallize important radiopharmaceuticals or their Re analogues in their physiologically relevant forms. For the present study, X-ray quality crystals of $Na_2[ReO(MAG_3)]\cdot 5H_2O$ (**1**) ($MAG_3$ = penta-anionic form of mercaptoacetyltriglycine) were obtained for structural determination. The structure of the dianion $[ReO(MAG_3)]^{2-}$ was compared with those of the mono-anions in $[Ph_4As][^{99}TcO(MAG_3H)]$ and $[Bu_4N][ReO(MAG_3H)]$.

### MATERIALS AND METHODS

NMR spectra were obtained at 300 MHz for $^1H$ and 75 MHz for $^{13}C$ with a General Electric QE-300 spectrometer in $Me_2SO$-$d_6$. Chemical shifts (ppm) were referenced to the solvent peak 2.49 ppm ($^1H$) and 39.9 ppm ($^{13}C$) vs. TMS (tetramethylsilane). Elemental analyses were performed by Atlantic Microlabs Inc., Atlanta, GA. FTIR spectra were recorded with a Bruker IFS 66 instrument. S-(Benzoyl)thioacetyltriglycine[8] and $ReOCl_3(Me_2S)(OPPh_3)$[9,10] were prepared by literature procedures.

**$Na_2$[ReO(mercaptoacetylglycylglycylglycine)]·$5H_2O$ (1).** SBzMAG$_3$ (0.31 g, 0.85 mmol) was dissolved in 63% MeOH/$H_2O$ (16 mL) and the pH was brought to 8 with 1 N NaOH. $ReOCl_3(Me_2S)(OPPh_3)$ (0.55 g, 0.85 mmol) was added to give a green suspension, which was heated to 65-70 °C. As the reaction proceeded, the pH was maintained at 8 by dropwise addition of 1 N NaOH. The green suspension gradually cleared to give an orange solution after 1 h. The solution was cooled to room temperature, filtered and extracted twice with $CHCl_3$. Acetone (100 mL) was added to the MeOH/$H_2O$ solution. On standing at 5 °C overnight, the solution deposited orange microcrystals, which were collected, washed with acetone and air dried. Yield: 0.20 g (39%). X-ray quality crystals were obtained by slow diffusion of EtOH into a saturated solution of the complex in $H_2O$. Anal. Calcd for $C_8H_{18}Na_2N_3O_{11}SRe$: C, 16.11; H, 3.04; N, 7.04. Found: C, 16.26; H, 2.99; N, 6.98. $^1H$ NMR: 4.61, 4.30 (d & d, 2H, J = 16 Hz $NCH_2$), 4.45, 4.09 (d & d, 2H, J = 18 Hz $NCH_2$), 4.15, 4.02 (d & d, 2H, J = 16 Hz $NCH_2$), 3.75, 3.58 (d & d, 2H, J = 17 Hz $SCH_2$). $^{13}C$ NMR: 192.56 (CO), 191.58 (CO), 188.48 (CO), 175.07 (CO), 60.30 ($NCH_2$), 56.17 ($NCH_2$), 53.62 ($NCH_2$), 38.84 ($SCH_2$). FTIR in KBr: 992 $cm^{-1}$ [Re=O].

**X-ray Crystallography.** An orange prism of **1** having approximate dimensions of 0.32 x 0.40 x 0.50 mm was used for room temperature data collection on a Siemens P4 diffractometer. The crystal system and cell dimensions were determined by automatic indexing of 25 centered reflections. High angle cell constants were obtained from 25 reflections >20° in 2θ at the conclusion of the data collection. Three check reflections were measured every 48 reflections and there was no significant deviation in intensities. Intensities were corrected for Lorentz and monochromator polarization effects, and a semi-empirical absorption correction was applied based on azimuthal scans of 5 reflections. The structure was solved successfully in the space group P $\bar{1}$ by Patterson methods, and all non-hydrogen atoms were refined anisotropically by full-matrix least-squares procedures using SHELXTL PLUS (VMS). The water hydrogen atoms were located from difference maps, and the methylene hydrogen atoms were generated at calculated (*d*(C-H) = 0.96 Å) positions. All hydrogen atoms were constrained during refinement using a riding model with isotropic thermal parameters fixed at 0.08. Crystallographic data are summarized in Table I.

**Table I.** Crystallographic Data for $Na_2[ReO(MAG_3)]\cdot5H_2O$ (**1**)

| | |
|---|---|
| chemical formula | $C_8H_{18}Na_2N_3O_{11}ReS$ |
| fw | 596.5 |
| space group | P $\bar{1}$ |
| Z | 2 |
| a (Å) | 8.907 (4) |
| b (Å) | 9.935 (4) |
| c (Å) | 11.610 (3) |
| α (deg) | 72.64 (2) |
| β (deg) | 79.32 (3) |
| γ (deg) | 64.65 (3) |
| volume ($Å^3$) | 884.2 (5) |
| $d_{calcd}$ ($g/cm^3$) | 2.240 |
| λ (Å) | 0.71073 (Mo Kα) |
| μ ($mm^{-1}$) | 7.183 |
| T (K) | 298 |
| min./max. transmission | 0.045 /0.081 |
| R (%) | 4.10 |
| $R_w$ (%) | 5.41 |

### RESULTS

Re complexes with the mercaptoacetyltriglycine ligand and its derivatives have generally been isolated as salts of large organic cations that can be extracted from acidified aqueous reaction

**Table II.** Atomic Coordinates (x$10^4$) and Equivalent Isotropic Displacement Coefficients (Å$^2$ x $10^3$) for $Na_2[ReO(MAG_3)]\cdot 5H_2O$ (**1**)

| atom | x | y | z | U(eq) |
|---|---|---|---|---|
| Re | 250(1) | 2057(1) | 639(1) | 20(1) |
| S | -2505(2) | 3689(2) | 530(1) | 32(1) |
| N(1) | 101(6) | 2401(6) | -1126(4) | 25(2) |
| N(2) | 2487(6) | 2044(6) | -40(5) | 27(2) |
| N(3) | 673(6) | 3169(6) | 1677(4) | 24(2) |
| O(1) | 388(7) | 310(5) | 1405(4) | 37(2) |
| O(2) | -1398(6) | 3045(6) | -2745(4) | 34(2) |
| O(3) | 4388(6) | 1831(7) | -1654(5) | 38(2) |
| O(4) | 2790(6) | 3261(6) | 2456(5) | 41(2) |
| O(5) | -1737(6) | 3485(6) | 4522(4) | 34(2) |
| O(6) | -427(8) | 1486(5) | 3727(5) | 45(2) |
| C(1) | -2885(8) | 3442(9) | -861(6) | 35(3) |
| C(2) | -1335(7) | 2959(6) | -1673(5) | 23(2) |
| C(3) | 1688(8) | 1903(8) | -1840(6) | 30(2) |
| C(4) | 3010(8) | 1926(7) | -1183(5) | 27(2) |
| C(5) | 3449(7) | 2225(8) | 722(6) | 29(2) |
| C(6) | 2274(8) | 2926(7) | 1716(5) | 27(2) |
| C(7) | -511(8) | 3933(7) | 2538(5) | 25(2) |
| C(8) | -898(7) | 2854(7) | 3670(5) | 25(2) |
| Na(1) | 531(3) | -1087(3) | 3885(2) | 33(1) |
| Na(2) | -4352(3) | 3221(3) | 4740(2) | 33(1) |
| O(7) | -4706(6) | 4211(6) | 6529(4) | 35(2) |
| O(8) | 3289(7) | -892(6) | 3731(5) | 42(2) |
| O(9) | -4060(7) | 1930(7) | 3173(5) | 46(2) |
| O(10) | -2236(7) | -993(6) | 4550(5) | 48(3) |
| O(11) | -7310(6) | 3791(6) | 4860(5) | 39(2) |

* Equivalent isotropic U defined as one third of the trace of the orthogonalized $U_{ij}$ tensor.

**Table III.** Selected Bond Distances (Å) and Angles (deg) for $Na_2[ReO(MAG_3)]\cdot 5H_2O$ (**1**)

| | | | |
|---|---|---|---|
| | Bond distances (Å) | | |
| Re-O(1) | 1.659 (5) | Re-N(2) | 1.998 (6) |
| Re-S | 2.292 (2) | Re-N(3) | 2.033 (7) |
| Re-N(1) | 1.995 (5) | | |
| | Bond angles (deg) | | |
| S-Re-O(1) | 108.9(2) | N(3)-Re-O(1) | 112.0(2) |
| S-Re-N(1) | 82.2(1) | Re-S-C(1) | 98.7(2) |
| S-Re-N(2) | 139.5(1) | Re-N(1)-C(2) | 124.8(4) |
| S-Re-N(3) | 92.5(1) | Re-N(1)-C(3) | 115.9(4) |
| N(1)-Re-N(2) | 78.0(2) | Re-N(2)-C(4) | 120.1(5) |
| N(1)-Re-N(3) | 135.1(2) | Re-N(2)-C(5) | 117.3(4) |
| N(2)-Re-N(3) | 77.9(2) | Re-N(3)-C(6) | 116.2(4) |
| N(1)-Re-O(1) | 111.8(3) | Re-N(3)-C(7) | 125.7(5) |
| N(2)-Re-O(1) | 111.2(2) | Re-O(1)-Na(1) | 130.9(3) |

solutions into organic solvents.[7,11] The complexes isolated using this procedure are monoanionic, with the carboxyl group protonated. However, ligand exchange of $ReOCl_3(Me_2S)(OPPh_3)$ with $N_3S$ ligands has proven to be a very clean process, and dianionic species can be

(micro)crystallized directly from the reaction solution by adding acetone. See also the synthesis of $Na_2[ReO(MAG_2\text{-}AMS)]\cdot 3H_2O$[12] ($MAG_2AMS$ = penta-anionic form of mercaptoacetylglycylgylcyl-aminomethanesulphonate). Once the sodium salt of $[ReO(MAG_3)]^{2-}$ was isolated in pure form, X-ray quality crystals were readily obtained by diffusion of EtOH into a saturated solution of the salt in $H_2O$. The advantage of this procedure was that the complex could be isolated in the same protonation state present in vivo.

Final atomic coordinates and selected bond distances and angles for $Na_2[ReO(MAG_3)]\cdot 5H_2O$ (**1**) appear in Tables II and III, respectively. A perspective drawing of the structure of the dianion of **1** is presented in Figure 1.

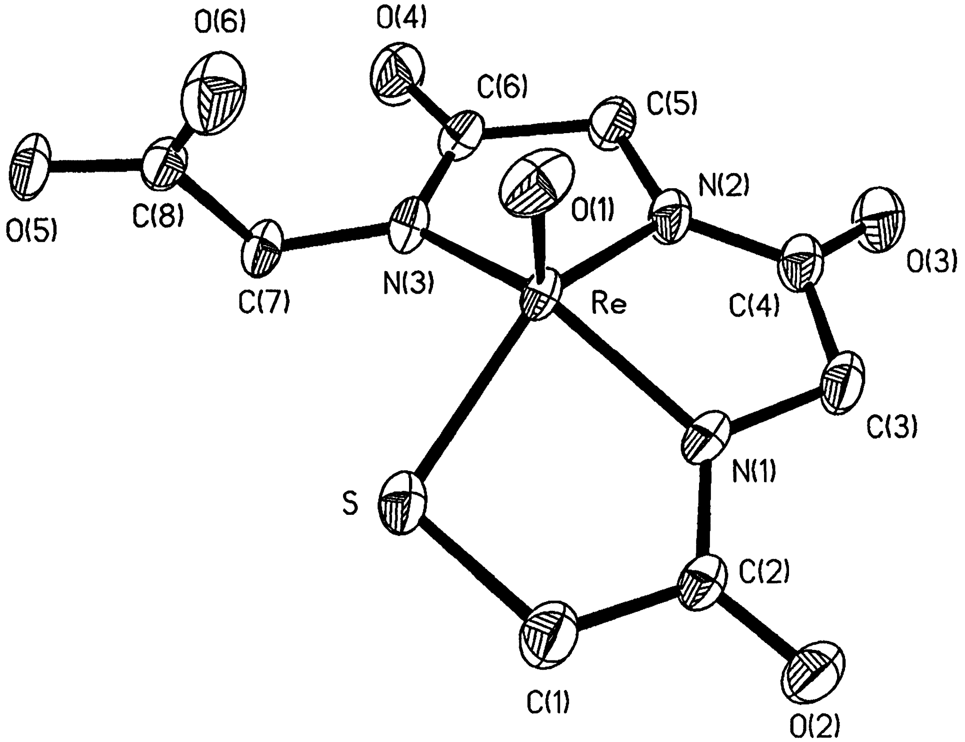

**Figure 1.** Perspective drawing of $[ReO(MAG_3)]^{2-}$ (dianion of **1**) with 50% probability for the thermal ellipsoids.

The coordination geometry is distorted square pyramidal with Re displaced 0.74 Å from the ligand coordination plane (S, N(1), N(2), N(3) mean deviation = 0.01 Å) toward the apical oxo ligand. The geometric parameters of the metal coordination sphere (i.e., bond distances and angles) (Table III) are nearly identical to those observed in $[Ph_4As][^{99}TcO(MAG_3H)]$.[5,6] Although the Re=O stretching frequency is shifted from 992 $cm^{-1}$ in **1**, to 975 $cm^{-1}$ in $[Bu_4N][ReO(MAG_3H)]$[illegible],[7] the Re-O(1) distance (1.659 (5) Å) in **1** is not significantly different from the Re-O(1) distance (1.68 (1) Å) in $[Bu_4N][ReO(MAG_3H)]$. IR data have not been reported for $[Ph_4As][^{99}TcO(MAG_3H)]$. The most significant difference within this series of complexes is the *anti* conformation in $[Bu_4N][ReO(MAG_3H)]$. Both **1** and $[^{99}TcO(MAG_3H)]^-$ have a *syn* conformation. The difference in conformation between $[Ph_4As][^{99}TcO(MAG_3H)]$[5,6] and $[Bu_4N][ReO(MAG_3H)]$[7] has previously been attributed to differences in crystal packing forces.[11] Both the conformation and orientation (N(3)-C(7)-C(8)-O(5) (161.2°) and (N(3)-C(7)-C(8)-O(6) (-20.9°) torsion angles) of the carboxylic acid group in $[Ph_4As][^{99}TcO(MAG_3H)]$ are apparently the result of intermolecular H(carboxyl)-bonding forces. However, the structures of the dianion of **1** and the monoanion $[^{99}TcO(MAG_3H)]^-$ are remarkably similar despite the difference in the nature of the cation(s) and protonation state of the (di)anions. Even the N(3)-C(7)-C(8)-O(5) (169.6°) and (N(3)-C(7)-C(8)-O(6) (-10.9°) torsion angles of **1** are similar to those of $[Ph_4As][^{99}TcO(MAG_3H)]$ (161.2 and -20.9°, respectively), although in **1** the carboxyl group is deprotonated and the carboxylate oxygen atoms (O(5) and O(6)) are coordinated to Na.

In the crystal lattice of 1, Na(1) is surrounded by three carboxylate oxygen atoms (O(6), O(6a) symmetry position (-x, -y, 1-z), and O(5a) symmetry position (-x, -y, 1-z)), the oxo group (O(1)), two water oxygen atoms (O(8) and O(10)) and a carbonyl oxygen atom (O(2a) symmetry position (-x, -y, -z)). Na(2) is surrounded by one carboxylate oxygen atom (O(5)) and five water oxygen atoms (O(7), O(7a) symmetry position (-1-x, -y, -z), O(8') symmetry position (-x, 1-y, 1-z), O(9) and O(11) (Figure 2). Na(1) is bridged to Na(2a) symmetry position (-x, -y, 1-z) by O(8) and O(5a) and to Na(1a) symmetry position (-x, -y, 1-z) by O(6) and O(6a). Na(2) is bridged to Na(1a) by O(5) and O(8a) and to Na(2b) by O(7) and O(7a). Since O(1) and O(6) are both coordinated to Na(1) and four of the seven atoms coordinated to Na(1) are oxo or carboxylate oxygens, the conformation and orientation of the carboxylate in 1 is clearly the result of intramolecular and intermolecular packing forces. Similar interactions between the oxo and sulphonate oxygen atoms, and Na were also observed in the structure of $Na_2[ReO(MAG_2\text{-}AMS)]\cdot 3H_2O$.[12]

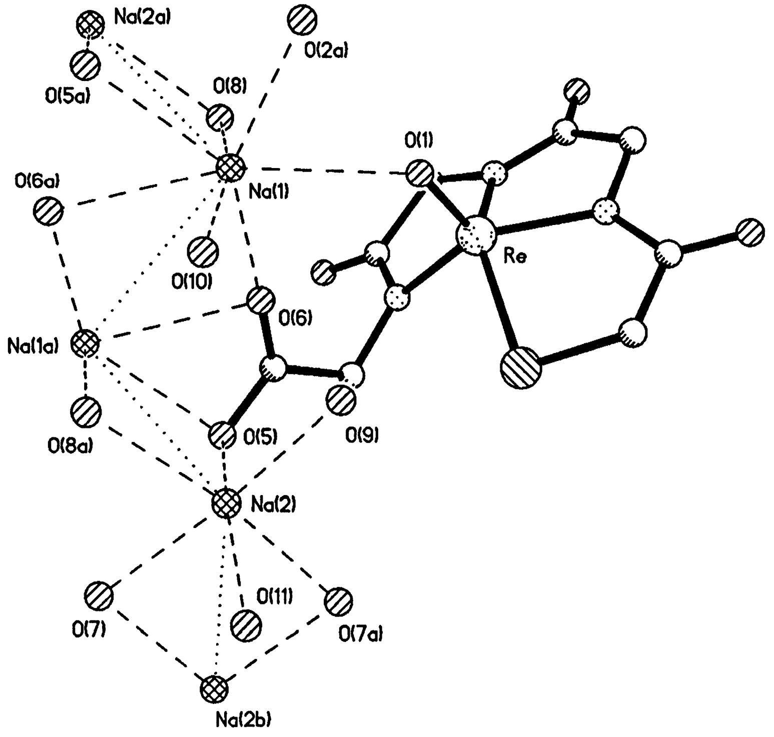

**Figure 2.** $Na^+$ coordination in the crystal lattice of $Na_2[ReO(MAG_3)]\cdot 5H_2O$ (**1**).

### DISCUSSION

The development of radiopharmaceuticals has largely been guided by empirical extrapolations and the minor modification of existing compounds with known pharmacokinetic parameters. Structure-distribution correlations have, therefore, often been made without adequate knowledge or consideration of the stereoelectronic properties of the radiopharmaceuticals. X-ray diffraction studies have provided useful solid-state structural data, but in many cases such information is of limited value because it may not be clear that the structures have relevance to the solution state. This is particularly true for the X-ray crystal structures of $[Ph_4As][^{99}TcO(MAG_3H)]$[5,6] and $[Bu_4N][ReO(MAG_3H)]$.[7]

Since the (ionized) carboxylate of Tc and Re complexes of mercaptoacetyltriglycine and its orientation are vital to the in vivo distribution of these molecules,[13,14] isolation of a salt of the dianion $[ReO(MAG_3)]^{2-}$ and the determination of its structure by X-ray diffraction was an important goal. The disodium salt was successfully crystallized; however, the X-ray crystal structure revealed that the overall molecular conformation is similar to that of the $^{99}Tc$ mono-anionic species. Coordination of the carboxylate oxygen atoms to the two sodium counterions imposes N(3)-C(7)-C(8)-O(5) and N(3)-C(7)-C(8)-O(6) torsion angles that are similar the $N\text{-}CH_2\text{-}C{=}O$ and $N\text{-}CH_2\text{-}C\text{-}OH$ torsion angles observed in $[Ph_4As][^{99}TcO(MAG_3H)]$.[5,6] The $N\text{-}CH_2\text{-}C\text{-}OH$ torsion angle is 21° in $[Ph_4As][^{99}TcO(MAG_3H)]$ (11° in $Na_2[ReO(MAG_3)]\cdot 5H_2O$) and has an unusually large torsion potential based on molecular mechanics calculations, while the angle is -66° in the energy-minimized structure of $[^{99}TcO(MAG^3H)]^-$.[11] Since the crystalline lattice, the charge, and even the metal center differ in these two cases this result indicates that the structure of $Na_2[ReO(MAG_3)]\cdot 5H_2O$ may have some population in solution even if it is different from the expected solution-phase structures of $[MO(MAG_3)]^{2-}$ (M = Tc, Re) complexes. Large non-coordinating cations may produce suitable crystals in which the structure of the dianion $[ReO(MAG_3)]^{2-}$ is different from the two general types already identified.

### ACKNOWLEDGMENT

We gratefully acknowledge financial support from the National Institutes of Health (Grant No. DK38842-08).

### REFERENCES

1. Taylor, A., Jr.; Clark, S.; Ball, T. *Clin. Nucl. Med.* 1994, **19**, 575.

2. Eshima, D.; Taylor, A., Jr. *Sem. Nucl. Med.* 1992, **22**, 61.

3. Vanderheyden, J. L.; Gofinch, S.; Srinivasan, A.; Su, F. M.; Venkatesan, P.; Woodhouse, C.; Sanderson, J.; Woods, M.; Wilkening, D.; Beaumier, P.; Reno, J.; Fritzberg, A. R. *J. Nucl. Med.* 1989, **30**, 793 (abstract).

4. Taylor, A., Jr.; Corrigan, P. L.; Garcia, E. V.; Folks, R.; Jones, M.; Cotsonis, G. *J. Nucl. Med.* submitted.

5. Nosco, D. L., Ph.D. Mallinkrodt, Inc. Unpublished data (cited with permission).

6. Nosco, D. L.; Manning, R. G. *J. Nucl. Med.* 1986, **27**, 939 (abstract).

7. Rao, T. N.; Adhikesavalu, D.; Camerman, A.; Fritzberg, A. R. *Inorg. Chim. Acta* 1991, **180**, 63.

8. Brandau, W.; Bubeck, B.; Eisenhut, M.; Taylor, D. M. *Appl. Radiat. Isot.* 1988, **39**, 121.

9. Wilkinson, G.; Grove, D. E. *J. Chem. Soc. A* 1966, 14.

10. Bryan , J. C.; Stenkamp, P. E.; Tulip, T. H.; Mayer, J. M. *Inorg. Chem.* 1987, **26**, 2283.

11. Hansen, L.; Cini, R.; Taylor, A., Jr.; Marzilli, L. G. *Inorg. Chem.* 1992, **31**, 2801.

12. Hansen, L.; Taylor, A., Jr.; Marzilli, L. G. *Metal-Based Drugs* 1994, **1**, 31.

13. Fritzberg, A. R.; Kasina, S.; Eshima, D.; Johnson, D. L. *J. Nucl. Med.* 1986, **27**, 111.

14. Rao, T. N.; Adhikesavalu, D.; Camerman, A.; Fritzberg, A. R. *J. Am. Chem. Soc.* 1990, **112**, 5804.

**Received: December 2, 1994 - Accepted: January 10, 1994 - Received in revised camera-ready format: January 27, 1995**

